# Decompressive craniectomy in paediatric traumatic brain injury: a systematic review of current evidence

**DOI:** 10.1007/s00381-018-3977-5

**Published:** 2018-09-13

**Authors:** Maddalena Ardissino, Alice Tang, Elisabetta Muttoni, Kevin Tsang

**Affiliations:** 1 Imperial College School of Medicine, Imperial College London, London, SW7 2AZ UK; 2 St. Helens and Knowsley Teaching Hospitals, Whiston Hospital NHS Trust, Liverpool, UK; 3 Department of Neurosurgery, Imperial College Healthcare NHS Trust, Charing Cross Hospital, London, UK

**Keywords:** Paediatric traumatic brain injury, TBI, Decompressive craniectomy, Surgery, Intracranial pressure, ICP, Outcomes, Management

## Abstract

**Introduction:**

Paediatric traumatic brain injury (pTBI) is one of the most frequent neurological presentations encountered in emergency departments worldwide. Every year, more than 200,000 American children suffer pTBIs, many of which lead to long-term damage.

**Objectives:**

We aim to review the existing evidence on the efficacy of the decompressive craniectomy (DC) in controlling intracranial pressure (ICP) and improving long-term outcomes in children with pTBI.

**Methods:**

A comprehensive search of the MEDLINE and EMBASE databases led to the screening of 212 studies, 12 of which satisfied inclusion criteria. Data extracted included the number and ages of patients, Glasgow Coma Scale scores at presentation, treatment protocols and short- and long-term outcomes.

**Results:**

Each of the nine studies including ICP as an outcome reported that it was successfully controlled by DC. The 6–12 month outcome scores of patients undergoing DC were positive, or superior to those of medically treated groups in nine of 11 studies. Mortality was compared in only two studies, and was lower in the DC group in both.Very few studies are currently available investigating short- and long-term outcomes in children with TBI undergoing DC.

**Conclusion:**

The currently available evidence may support a beneficial role of DC in controlling ICP and improving long-term outcomes.

## Introduction

### Background

Paediatric traumatic brain injury (pTBI) is one of the most frequent neurological emergencies affecting children throughout the world: ten million injuries lead to hospitalisation or death every year [1]. In the USA alone, approximately 230,000 children suffer a TBI every year, and these lead to even more severe and long-lasting neurological disabilities than those occurring in adolescents or adults [2]. Despite this, there is little evidence on which to base protocols for the management of pTBI as most of the treatment algorithms are founded on experience gained in adults and adapted on the basis of subtle differences in physiology and anatomy. However, since the introduction of the 2012 guidelines, a number of studies have been published concerning especially the surgical management of adult and pTBI, the results of which can be considered for future modifications of management protocols.

### Aims

The aim of this review is to summarise and assess current methods of surgically managing pTBI, concentrating on the use of decompressive craniectomy (DC) as a means of reducing intracranial pressure (ICP) in the short term, and improving rehabilitative outcomes in the long term. Although it is still a subject considerable debate, it has been shown that DC effectively decreases ICP and its fluctuations, and may increase cerebral perfusion pressure [3], it has also been shown to be more economically effective than medical management approaches such as the use of barbiturate-induced coma [4]. However, its impact on the clinical outcomes of TBI patients has yet to be fully ascertained. A systematic review was carried out with the aim of summarising the currently available evidence on the effect of the DC on ICP reduction in the short term, and rehabilitation outcomes in the long term.

This review considers the published evidence concerning the short- and long-term outcomes of DC in children pTBI, as well as the most important recent studies of its therapeutic role in adult patients.

## Methods

### Literature search

This systematic review was made following the guidelines proposed in the PRISMA statement [5]. The MEDLINE and EMBASE databases were searched using the terms ‘(paediatric traumatic brain injury) AND (decompressive craniectomy)’. Only studies published in English were considered.

Of the 617 studies published up to October 2017, we selected the randomised clinical trials (RCTs), case series or two-arm studies that involved patients aged < 18 years, included TBI patients who underwent DC to control ICP, and measured long-term (> 4 weeks) outcomes. Individual case reports were excluded, as were studies that did not provide quantitative data or were designed to answer different questions (e.g. those investigating complications or outcome predictions).

**Fig. 1:**
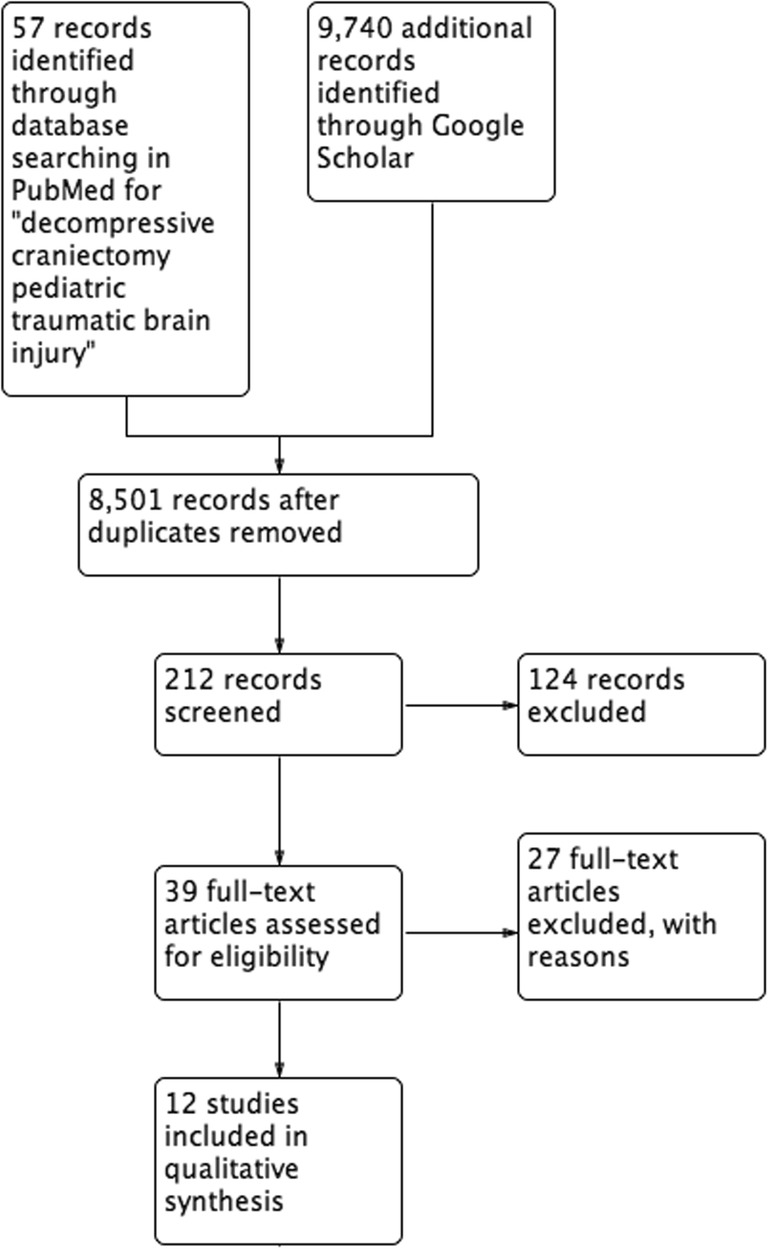
PRISMA flowchart of study selection

Twelve of the 212 screened studies satisfied our selection criteria (one RCT, and 11 case series involving a total of 260 patients), and were read in full and analysed by all of the authors of this review. The selection process is outlined in Fig. 1.

### Data analysis

The extracted data included the names of the authors and the year of study publication, the number and age ranges of the patients involved, Glasgow Coma Scale (GCS) scores at the time of presentation, treatment protocols, and short- and long-term outcomes and mortality. This information was summarised and reviewed by three reviewers independently (MA, EM and AT), and further evaluated by the senior author (KT). Two of the reviewers independently assessed the quality of the data using the GRADE scoring system of the *British Medical Journal* (BMJ) [6], and the risk of bias was analysed using the Cochrane risk of bias tool [7].

## Results

### Quality assessment

The only RCT that met the inclusion criteria [8] was judged to be at risk of bias on the basis of the Cochrane risk of bias tool because of incomplete information regarding the blinding of the outcome measurer. A number of studies enrolled only a few patients, and some did not fully describe outcomes, or assess and control for confounding factors. Overall, the number of patients included in the 12 studies analysed is extremely low, varying from 5 to 53 (median 17) per study. Table 1 shows the level of the quality of evidence for each study on the basis of the BMJ’s GRADE scoring system, and the risk of bias was assessed using RevMan [20].

**Table 1:** Quality of evidence assessment for the included studies

Author/year	Design	No. of patients/age	Limitations	Consistency	Directness	Other factors	Quality
Cho 1995 [9]	Case series	*n* = 17 2–12 months	No serious limitations	No important inconsistency	Direct	None	Moderate
Thomale 2010 [10]	Case series (retrospective)	*n* = 53 < 16 years	No serious limitations	No important inconsistency	Direct	None	Moderate
Taylor 2001 [8]	RCT	*n* = 27 1–18 years	No serious limitations	No important inconsistency	Direct	None	High
Hejazi 2002 [11]	Case series	*n* = 7 1–18 years	No serious limitations	No important inconsistency	Direct	Few data	Low
Figaji 2003 [12]	Case series	*n* = 5 5–12 years	Limitations	No important inconsistency	Direct	Few data	Low
Ruf 2003 [13]	Case series	*n* = 6 5–11 years	No serious limitations	No important inconsistency	Direct	None	Moderate
Josan 2006 [14]	Case series (retrospective)	*n* = 12 2–16 years	No serious limitations	No important inconsistency	Direct	Limited number of participants	Moderate
Kan 2006 [15]	Case series	*n* = 51 4 months–14 years	No serious limitations	No important inconsistency	Direct	Imprecise data	Low
Rutigliano 2006 [16]	Case series	*n* = 6 12–15 years	No serious limitations	No important inconsistency	Direct	Limited number of participants	Moderate
Skoglund 2006 [17]	Case series (retrospective)	*n* = 19 7–16 years	No serious limitations	No important inconsistency	Some uncertainty about directness	High risk of reporting bias	Low
Jagannathan 2007 [18]	Case series (retrospective)	*n* = 23 2–19 years	No serious limitations	No important inconsistency	Direct	High risk of reporting bias	Low
Guresir 2012 [19]	Case series	*n* = 34 0–18 years	No serious limitations	No important inconsistency	Some uncertainty about directness	Imprecise data	Very low

### Effect of DC on ICP

Of the 12 studies reviewed, nine included ICP as a short-term outcome. All nine reported that ICP was successfully reduced by DC and that the patients required fewer ICP control interventions. Two studies directly compared ICP control in patients receiving medical treatment (MT) and those undergoing DC. Cho et al. [9] found an 80% reduction in ICP in DC patients, which was greater than that observed in the MT group (*p* < 0.05), and the RCT by Taylor et al. [8] also found that DC led to better ICP control than MT, although the difference was not statistically significant (*p* = 0.057). The rates of ICP control achieved in all of the other studies [10, 12–16, 18] ranged from 69.4 to 100% (Table 2).

**Table 2:** Characteristics and outcomes of included studies

Author /year	Study	No. of patients/age	Treatment	Outcome measures	ICP results	Functional outcome
Cho 1995 [9]	Case series	*n* = 17 2–12 months	ICP > 30 mmHg treated with DC, *n* = 10 (unilateral or bilateral) MT, *n* = 7	ICP Mortality 6-month 6-year COS	80% reduction with DC (*p* < 0.05)	Mortality lower in DC group (0/10 vs 3/7; *p* < 0.05)
						Better 6-month 6-year COS in DC group (*p* < 0.05)
						Hearing preservation higher in DC group (*p* < 0.05)
Thomale 2010 [10]	Case series (retrospective)	*n* = 53 < 16 years Median GCS = 6.5 in DC group and 3 in MT group	Severe TBI patients presenting at the author’s centre, 14 DC and 39 MT	ICU stay ICP GOS	ICP control achieved in all DC patients. No report on ICP in MT patients	No significant difference in 12-month and long-term GOS
Taylor 2001 [8]	Retrospective controlled trial	*n* = 27 1–18 years	If high ICP, randomised to early DC (bitemporal craniectomy) or MT	ICP CPP ICU stay 6-month GOS	Better control with DC (*p* = 0.057)	6-month GOS ‘favourable’ in 14% of MT group vs 54% of DC group (*p* = 0.048)
Hejazi 2002 [11]	Case series	*n* = 7 1–18 years	DC if herniation or decorticate posturing (unilateral DC)	5-week GCS	N/A	All had GCS 15 after 5 weeks
Figaji 2003 [12]	Case series	*n* = 5 5–12 years Mean GCS 4.6 (3–9)	DC if GCS < 8 (unilateral with duraplasty floating flap)	ICP GOS	Full ICP control in 2 and moderate reduction of ICP in 2	All patients had GOS 4–5 at time of follow-up (14–40 months)
Ruf 2003 [13]	Case series	*n* = 6 5–11 years	DC performed in patients with ICP > 20 mmHg for > 30 min	ICP 6-month survival and 6-month neuro-logical follow-up	ICP normalised immediately in all cases	4 had no disability and 2 mild/moderate disability
Josan 2006 [14]	Case series (retrospective)	*n* = 12 Mean GCS 6.8 6 DC, 6 MT	DC or MT in patients with refractory high ICP post-TBI Mean time between TBI and DC 7 h	ICP 12-month GOS	Mean ICP after intervention 12.33 mmHg	100% survival in DC group, 66% survival in MT group
						6 months, 100% favourable GOS in DC group vs 50% in MT group
						Early intervention (may improve outcome)
Kan 2006 [15]	Case series	*n* = 51 4 months −14 years Mean GCS 4.6	DCs performed at the author’s institution between 1996 and 2005 ± mass lesion evacuation	ICP Mortality KOSCHI	69.4% had normal ICP after surgical intervention	31% died
						Mortality highest (5/6) in patients who underwent DC for ICP alone (no mass lesion)
						Mean follow-up KOSCHI 4.5
Rutigliano 2006 [16]	Case series	*n* = 6 12–15 years	DC if refractory high ICP	ICP FIM	ICP normalised in 5/6	6/6 had FIM indicating independence or minimal assistance at discharge
Skoglund 2006 [17]	Case series	*n* = 19 7–16 years	DC if GCS deterioration, herniation and refractory ICP	12-month GOS	N/A	3 patients GOS 5
						1 patient GOS 4
						1 patient GOS 3
						1 patient died
Jagannathan 2007 [18]	Case series (retrospective)	*n* = 23 2–19 years	DCs performed at the centre between 1995 and 2006	ICP GOS Likert QOL scale	83% ICP controlled with DC	7 died
						83% of the survivors returned to school
						Mean follow-up GOS 4.5, median 5
Guresir 2012 [19]	Case series	*n* = 34 (23 TBI) 0–18 years	DC performed in 23 TBI patients, 2 SAH, 3 ICH, 5 infarction and 3 other	Modified Rankin Score (favourable 0–2) Return to school	N/A	Favourable outcome in 40% of TBI patients
						30% did not return to school due to disability
						9th grade, 1
						10th grade, 5
						13th grade, 1

*DC* decompressive craniectomy, *MT* medical therapy, *ICP* intracranial pressure, *ICU* intensive care unit, *GOS* Glasgow Outcome Score, *COS* Children’s Outcome Score, *CPP* cerebral perfusion pressure, *FIM* Functional Independence Measure, *QOL q*uality of life, *KOSCHI* King’s Outcome Scale for Closed Head Injury, *SAH* sub-arachnoid haemorrhage, *ICH* intra-cerebral haemorrhage

### Effect of DC on mortality

Only two of the studies compared mortality among the patients undergoing DC and those receiving MT. Cho et al. [9] found that mortality was significantly lower in the patients who underwent DC (0/10 vs 3/7; *p* < 0.05), and Josan et al. [14] recorded higher survival rates (100% vs 66%) (Table 2).

### Effect of DC on long-term outcomes

The method of assessing long-term outcomes varied: the most widely used scoring system was the original Glasgow Outcomes Scale (GOS) or the Extended Glasgow Outcomes Scale (GOS-E), and several studies included other systems (the Functional Independence Measure, Children’s Outcome Score, King’s Outcome Scale for Closed Head Injury and quality of life scales). These systems generally assess functional ability and independence, return to school and performance in doing everyday activities but, given their heterogeneity, we qualitatively compared the results by dividing them into positive or negative functional outcomes. Complete recovery, or a mild disability that does not interfere with independence or activities, was regarded as a positive outcome, and severe disability, dependency, vegetative state and mortality as negative outcomes.

Ten studies reported positive outcomes in the patients who underwent DC [8, 9, 11–19]. Four directly compared the follow-up GOS scores of the patients who underwent DC with those of the patients receiving MT [8–10, 14] (Table 2). Cho et al. [9] found that the scores assigned between 6 months and 6 years after a TBI were significantly better in the patients who underwent DC (*p* < 0.05). This is in line with the results of the retrospective study of Josan et al [14]: 6 months after their TBIs, all of the DC patients were assigned a favourable GOS score as against 50% of those who received MT. Taylor et al. [8] also found that the DC group had a higher incidence of favourable GOS scores after 6 months (54% vs 14% in the MT group), but Thomale et al. [10] did not find any significant difference in long-term GOS scores between the two groups.

## Discussion

The use of DC to treat high ICP in paediatric and adult TBI patients has long been a subject of debate. The recommendations for surgery in children are even less clear than those included in the adult guidelines because of the severe lack of clinical evidence [21]. As highlighted in this review, it has been consistently found that ICP is well controlled by DC in children with TBI in the short term, but the correlation between successful ICP control and long-term clinical outcomes is more questionable. Furthermore, the existing evidence is derived from studies with high risk of bias, and containing low patient numbers. The current guidelines [21] recommend DC when performed together with other surgical procedures such as haemorrhage evacuation, or if there is strong suspicion of herniation, but its use as a stand-alone procedure to relieve ICP in patients without herniation is limited to those with intracranial hypertension (> 25 mmHg) showing signs of neurological deterioration, or high ICP refractory to optimal MT.

The findings of the retrospective case series and the RCT reviewed generally indicate that DC has a positive effect in controlling ICP, though the quality of evidence is generally low. Hejazi’s [11] study reported a full recovery in five out of six patients who underwent the procedure, Figaji et al. [12] similarly reported notable improvements in neurological function in a cohort of patients on whom DC was performed following neurological deterioration, and similar findings were reported by Ruf et al. [13] and others. The only RCT was conducted by Taylor et al. [8], who compared the outcomes of DC and MT in respectively 27 children with refractory an ICP of > 30 mmHg and found that they were much worse in the children receiving MT. However, only two studies compare ICP control directly between patients receiving MT and those undergoing DC. Mortality was only analysed in two studies with low patient numbers [9, 14], of 12 and 17 respectively. More studies looked at long-term outcomes; however, outcome scales used to compare these were variable; it is therefore hard to draw general conclusions from them.

Although adult studies do not provide direct evidence concerning the paediatric use of DC, it is important to consider their results as supplementary information not least because there are more high-quality studies referring to adults. The DECRA study (decompressive craniectomy in diffuse traumatic brain injury) found that patients with refractory intracranial hypertension (> 20 mmHg) who underwent DC required shorter ICU stays and fewer interventions to control ICP, but experienced worse long-term clinical outcomes [22]. However, questions have been raised about the potential bias of this study mainly because the randomisation process led to unbalanced cohorts with discrepancies in the severity of TBI (greater in the DC group) and GCS scores upon admission; furthermore, it has been pointed out that the definition of refractory raised ICP (> 20 mmHg for > 15 min) does not reflect clinical practice [23]. On the other hand, the RESCUE-ICP (trial of decompressive craniectomy for traumatic intracranial hypertension) trial found that the use of DC in adult patients with an ICP of > 25 mmHg was associated with fewer deaths and cases of severe disability than medical management, although it was also associated with a higher incidence of patients experiencing a vegetative state. There was no difference in the incidence of ‘good outcomes’ between the two groups [24]. Table 3 summarises the results of the most important recent studies of adults undergoing DC.

**Table 3:** Characteristics and outcomes of recent important studies in the adult population

Author/year	Study type no. Patients Age	Treatment	Outcome measures	Results, outcomes	Study quality and bias
Cooper 2011 [22]	Randomised clinical trial *n* = 155	Patients with ICP > 20 mmHg for > 15 min Randomly allocated to DC or MT	ICP 6-month GOS-E	ICP DC group had fewer hours with high ICP than MT group (*p* < 0.001) DC group had fewer days in ICU DC group had fewer ICP control interventions Outcomes DC group had worse GOS-E (OR 1.84, CI 1.05–3.24; *p* = 0.03) DC group at greater risk of unfavourable outcomes (death, severe disability and vegetative state), OR 2.21, CI 1.14–4.26; *p* = 0.02	ICP threshold does not reflect clinical guidelines for DC
					Mismatch in severity of TBI between DC and MT group
Timofeev 2006 [25]	Retrospective observational study *n* = 49 Age 9–67 years	DC, bilateral or unilateral in patients with persistently high ICP	6-month GOS and SF-36 QOL questionnaire	Outcomes at 6 months, 30 (61.2%) had good outcomes, 10 (20.4%) had severe disability and 9 (18.4%) died	No comparison with untreated patients
					No randomisation or control for confounders
Hutchinson 2016 [24]	Randomised clinical trial *n* = 408 Age 10–65 years	Refractory ICP > 25 mmHg Randomly allocated to DC or MT	Mortality 6-month GOS	ICP DC group had fewer hours with high ICP than MT group (*p* < 0.001) Outcomes DC group had lower mortality rate MT group had lower severe disability rate Rates of good recovery and moderate disability were the same	

*DC* decompressive craniectomy, *MT* medical therapy, *ICP* intracranial pressure, *GOS* Glasgow Outcome Score

## Conclusions

This review aims to summarise the presently available evidence in the treatment of paediatric traumatic brain injury using decompressive craniectomy versus medical treatment. The evidence considered in this review indicates a possible benefit in use of DC in patients with pTBI for reducing high ICP (> 25 mmHg) that is refractory to medical treatment. However, the quality of evidence remains extremely low, and there is very little evidence from RCTs to indicate whether this correlates with long-term benefits in the paediatric population. The findings of retrospective studies generally indicate a beneficial effect with improved long-term neurological recovery, but they are sometimes inconsistent and their quality varies because of differences in the patient age, the criteria for and timing of surgery, injury factors, rating scales used and the use of concomitant medical treatment. Overall, though available evidence unanimously indicates a short-term benefit in using DC to reduce ICP and mortality, and possible long-term rehabilitative improvement, the assessment of evidence quality carried out highlights the lack of evidence in the field, and further high-quality studies on larger patient numbers are certainly required.
